# A Case of Squint Correction With Toric Implantable Phakic Contact Lens (IPCL) Implantation: A Novel Approach to High Myopia With Sensory Exotropia

**DOI:** 10.7759/cureus.64175

**Published:** 2024-07-09

**Authors:** Shreya Gandhi, Radhika Paranjpe, Parikshit Gogate, Kalpita B Goli, Khushboo Goyal

**Affiliations:** 1 Ophthalmology, Dr. D.Y. Patil Medical College, Hospital and Research Centre, Pune, IND; 2 Ophthalmology, Dr. Gogate’s Eye Clinic, Pune, IND; 3 Ophthalmology, Dr. D Y. Patil Medical College, Hospital and Research Centre, Pune, IND

**Keywords:** refractive procedure, myopia, resection, recession, ipcl, exotropia

## Abstract

A 39-year-old male patient presented to the outpatient department (OPD) with chief complaints of outward deviation of the right eye (RE) since six months of age, associated with a diminution of vision in the same eye since childhood. He had a history of spectacle use for distance for the past eight years. He was thoroughly evaluated in the OPD and diagnosed with RE high myopia with sensory exotropia. Lateral rectus (LR) recess with medial rectus (MR) resection with implantable phakic contact lens (IPCL) implantation was planned and executed for the patient. He was started on topical steroids, antibiotics, non-steroidal anti-inflammatory drugs (NSAIDs), and lubricants post-surgery and was closely followed up. IPCL implantation with strabismus correction surgery is not usually performed together, especially in a young population, considering the risks involved, but it was performed in our case and gave satisfactory results. The patient had a significant improvement in visual acuity, and the correction of deviation was substantial.

## Introduction

High myopia is a crucial cause of loss of vision and is a refractive error of spherical equivalent greater than −6D (diopters) or an axial length of more than 26.5 mm. In a recent study from AIIMS, New Delhi, done on school-going children in North India, the prevalence of high myopia was found to be 1.5% [1]. Implantable phakic contact lens (IPCL) refers to the implantation of a lens in the eye without the removal of the normal crystalline lens. It is made up of acrylic and is a small, soft, single-piece, foldable posterior chamber intraocular lens (IOL). Preservation of accommodation is a distinct advantage over other modalities of treatment, such as clear lens extraction. It is inserted through a small incision and placed just in front or behind the iris [2].

Exotropia is the outward deviation of the eye and may be congenital or acquired. Risk factors for exotropia include neurological disorders, genetic disorders, positive family history, and refractive errors, among a few. Incomitant squint can be either paralytic or restrictive. Concomitant exodeviation can be classified as congenital, primary, sensory, or consecutive. Infantile exotropia is characterized by the onset of exodeviation in the first six months of life and has no spontaneous resolution [3]. While a variety of treatment modalities are available for the management of high myopia, IPCL surgery is a fairly reasonable alternative, especially in patients with very thin corneas where refractive procedures are contraindicated.

## Case presentation

A 39-year-old male patient came to the outpatient department with complaints of outward deviation of the right eye (RE) since six months of age, which was noticed by his mother and associated with a diminution of vision in the RE since childhood and was gradual and painless in nature. He denied any complaints of associated double vision. The patient had a history of wearing spectacles for distances for eight years, with no history of any ocular surgery or trauma in the past. He had no systemic illness like diabetes mellitus (DM), hypertension (HTN), ischemic heart disease (IHD), and asthma and was a non-smoker. The patient was born of a full-term normal delivery (FTND) with no history of neonatal intensive care unit (NICU) stay or any complications in the perinatal period.

**Figure 1:**
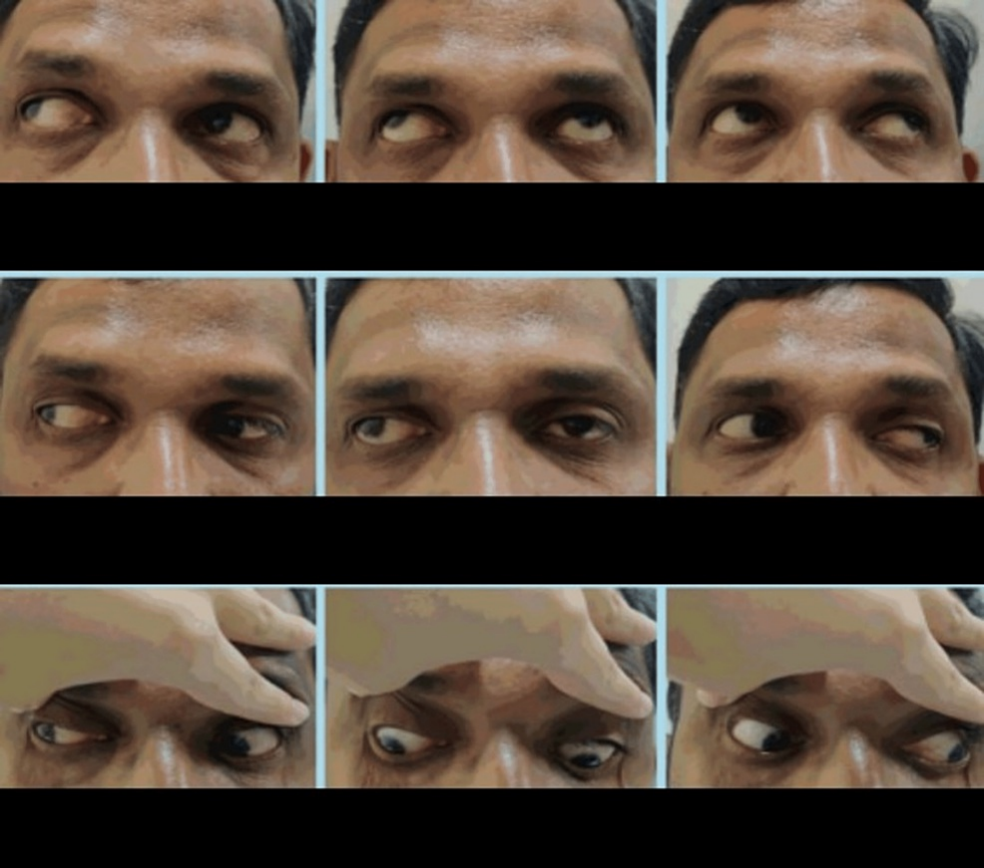
Image depicting extraocular movements in all gazes and pseudoproptosis of the right eye

**Figure 2:**
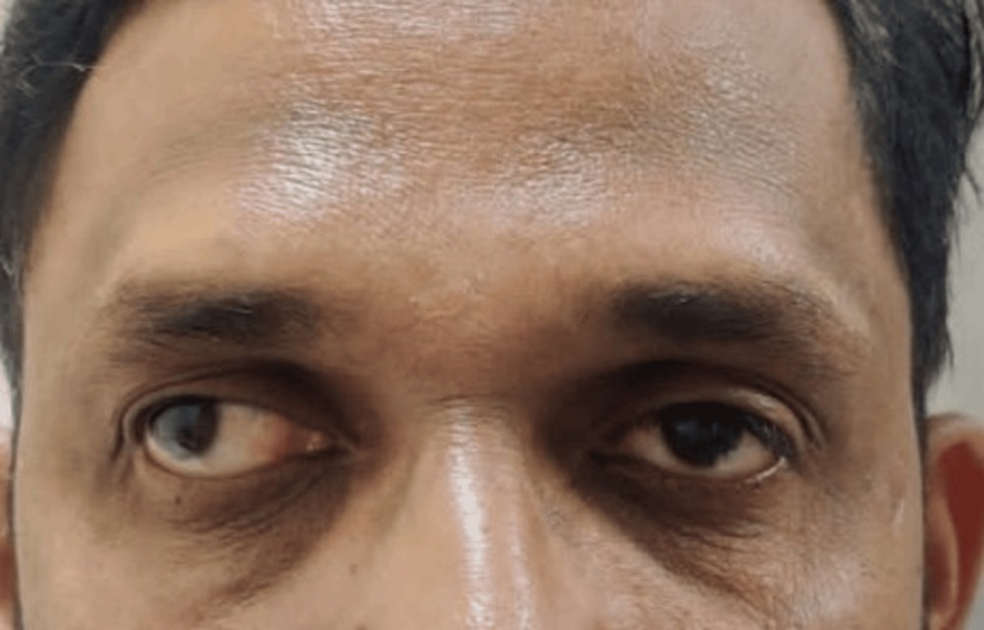
Image depicting an exotropia of approximately 30 degrees in the right eye

On ocular examination, his visual acuity on the Snellen chart was 1/60, improving to 6/18 in the RE with −11D with cylinder −2.50 at the 65-degree axis, and 6/60, improving to 6/6 in the left eye (LE) with −2D with cylinder −0.50 at the 80-degree axis, with near and color vision checked with the Ishihara chart to be within normal limits (WNL). Extraocular movements were full, free, and painless, with evidence of pseudoproptosis in the RE (Figure 1). On the Hirschberg corneal reflex test, there was 30 degrees of exotropia (Figure 2). On the cover uncover test, an alternating exotropia with LE dominance was inferred. The prism bar cover test showed 60 prism diopters of exotropia in the RE in primary gaze, upgaze, and downgaze for distance, while the LE was orthophoric.

**Figure 3:**
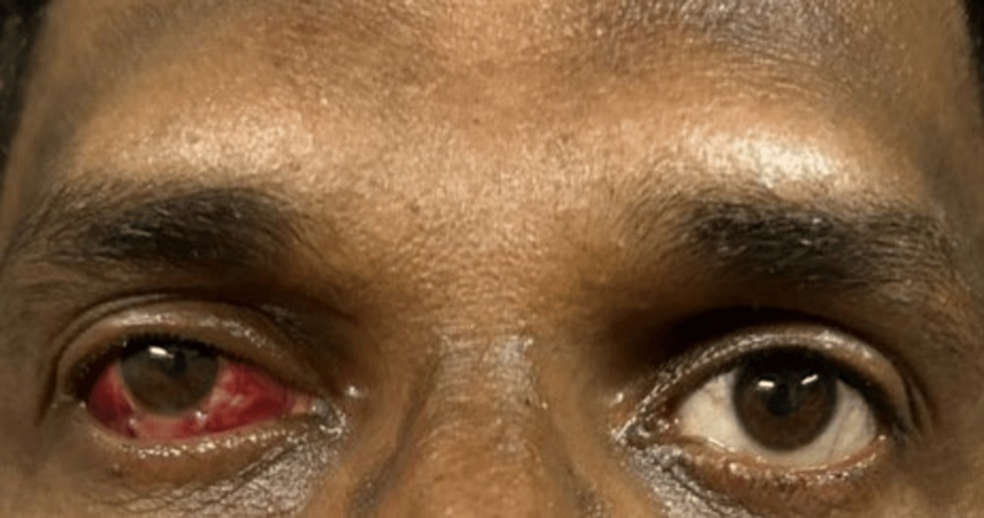
Image depicting a residual exotropia of approximately 15 degrees in the right eye on postoperative day 1, with upper and lower lid edema and subconjunctival hemorrhage

**Figure 4:**
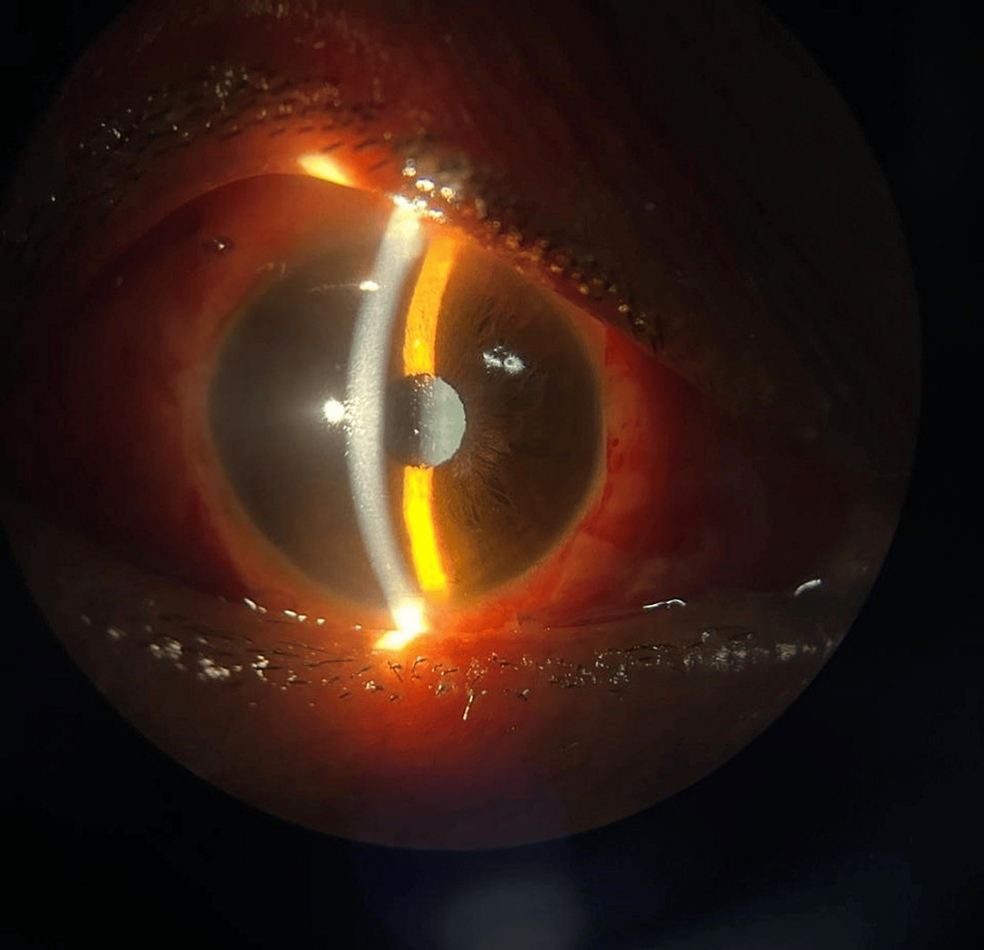
Image depicting slit lamp photograph of the right eye on postoperative day 1 with the aquaport of the IPCL V2.0 visualized centrally IPCL, implantable phakic contact lens

Slit lamp examination of both the eyes was WNL. Fundus examination was performed with a 90D lens and direct and indirect ophthalmoscope, which showed peripapillary atrophy in the RE, and the LE was WNL. Intraocular pressure using an applanation tonometer was measured to be 20 mmHg in the RE and 18 mmHg in the LE. Axial length was calculated using IOL Master 700, which was 28.02 mm in the RE and 24.42 mm in the LE. Pachymetry was performed for the patient, which revealed a central corneal thickness (CCT) of 450 microns in the RE and 480 microns in the LE. A diagnosis of RE high myopia with sensory exotropia was established, and the patient underwent a lateral rectus (LR) recession of 9 mm with medial rectus (MR) resection of 4 mm with IPCL implantation under local anesthesia, operated by one of the authors (PG). A toric IPCL V2.0 of the care group, which is a foldable, hydrophilic, acrylic IOL of −13.00D with cylinder +2.50 at a 150-degree axis, was implanted. On immediate postoperative day 1, the patient’s best corrected visual acuity (BCVA) in the operated eye was 6/18, he had a residual exotropia of 15 degrees, and the IOP measured on the non-contact tonometer was 20 mmHg in the RE (Figures 3, 4).

The patient was started on topical medications consisting of eye suspension prednisolone 1% once every three hours, eyedrop moxifloxacin 0.5% four times/day, eyedrop nepafenac 0.1% three times/day, and eyedrop carboxymethylcellulose 0.5% four times/day. The patient was adequately explained about the postoperative care and advised a close follow-up. On postoperative day 5, his BCVA in the RE was 6/9, and he had an exotropia of 10-15 degrees. He was consistently followed up for a month and had a satisfactory visual outcome, markedly decreased exodeviation, consistent intraocular pressure, and no signs of pupillary block glaucoma. He had a vision of 6/6 in the LE with the already mentioned correction, and it was prescribed on subsequent follow-up.

## Discussion

Myopia is defined as a refractive error where parallel rays of light from infinity are focused in front of the eye with accommodation being at rest. High myopia is defined as myopia of more than −6.0D and is associated with potentially blinding complications, such as glaucoma, retinal detachment, and myopic macular degeneration [1]. It has become a major public health problem globally, with a prediction of up to 50% of the world population to be myopic by 2050.

Various treatment modalities available include spectacles, contact lenses, orthokeratology, refractive procedures, and the relatively new concept of phakic IOL implantation. The indications depend on an approximate range of myopia. IPCL can be used for myopia of −3 to −15D with up to 2.5D astigmatism at the spectacle plane, whereas LASIK is for myopia of up to 8D. Radial keratectomy corrects low to moderate myopia of −2 to −6D, and similar to photorefractive keratectomy, clear lens extraction can be done for very high myopia −16 to −18D [4].

Phakic IOL implantation has been established as an effective alternative for ametropia correction, allowing a greater range of correction in comparison to keratorefractive procedures. However, the invasiveness may predispose the patients to complications, such as cataracts, a rise in IOP, endophthalmitis, and endothelial cell loss. Indications of implantable phakic IOLs include high myopia of greater than −8 to −10D or those unfit for conventional laser treatment (LASIK, PRK) with stable refractive power for one year, among a few others. Due to high myopia of −11D and a corneal thickness of 450 microns in the RE in our patient, a corneal refractive procedure wasn’t recommended, and an IPCL was tried and successfully executed. Endothelial cell density (ECD) loss with ICL in a study by Alfonso et al. had a mean ECD loss ranging from 0.3% to 7.8% [5].

A majority of surgeons globally can now easily execute photoablation refractive procedures; however, there are several consequences to be aware of, including high index aberrations, postoperative ectasia, refractive regressions, and ocular surface diseases, including dry eye. The introduction of IOLs, which can be implanted in the posterior chamber or the anterior chamber (AC) and can be iris-fixated or angle-supported, has expanded refractive surgery beyond laser technology [6,7]. Multiple studies have assessed several PIOL models (such as Artisan, Artiflex, and implantable collamer lens); however, there are only two papers that go into deeper detail about the implanted phakic contact lens known as IPCL (Care Group, India). In a three-year follow-up of 30 eyes, Vasavada et al. observed an endothelial cell loss of 9.73%, satisfactory refractive outcomes, and no complications [7]. After implanting the IPCL V1.0 model, Sachdev et al. evaluated 134 eyes for a year and came to the conclusion that IPCL is a safe and efficient procedure for the correction of myopia and myopic astigmatism [6].

IPCLs are injectable, foldable, and intended for placement behind the iris, with the haptic zone resting on the ciliary sulcus. It can correct hypermetropia from +1.0 to +15.0D and myopia from −1.00 to −30.00D within its dioptric power range. For individuals with extreme myopia or thin corneas, it can be challenging to offer independence from glasses; nonetheless, PIOLs are a great option for them, as laser corneal refractive procedures are not advised. PIOLs preserve the accommodative function [2].

Exotropia is defined as the outward deviation of either one or both eyes, which can be present intermittently or constantly. Incomitant squint can either be paralytic or restrictive. Concomitant exodeviation can be categorized as congenital, primary, sensory, or consecutive. Poor fusional reserves and a substantial angle constant exodeviation of more than 35 prism diopters are features of congenital exotropia. In these patients, the incidence of myopia is significantly higher than that of intermittent exotropia. Sensory exotropia is more common when unilateral vision loss occurs in infancy or adulthood. It could occur consecutively in any macular pathology, unilateral aphakia, optic atrophy, unilateral medial opacity, or anisometropia. When there is a documented progressive loss of fusional control, and the exodeviation lasts for more than 50% of the waking hours, surgery may be necessary [3].

Thus, both unilateral high myopia and exotropia can lead to poor stereopsis and amblyopia and should be managed with a modality suitable to the condition and the patient’s compliance to avoid major complications. Diplopia can also be one of the major postoperative complications and should be explained appropriately to the patients.

## Conclusions

Although IPCL implantation with strabismus correction surgery is not usually performed together, it was attempted in our patient and gave highly gratifying results. The patient had a remarkable improvement in visual acuity, and the correction of deviation was substantial, with no postoperative complications. Although the recurrence of strabismus is unpredictable, a timely surgery could prevent amblyopia.

Thus, from our inference, patients with high myopia with strabismus should be adequately managed to avoid amblyopia and restore binocularity.
